# Detection of SARS-CoV-2 RNA by Reverse Transcription-Polymerase Chain Reaction (RT-PCR) on Self-Collected Nasal Swab Compared With Professionally Collected Nasopharyngeal Swab

**DOI:** 10.7759/cureus.25618

**Published:** 2022-06-03

**Authors:** Nusrat Mannan, Ruksana Raihan, Ummey Shahnaz Parvin, Sheikh Mohammad Fazle Akbar, Md Selim Reza, Shafiqul Islam, Joy Kundu, Abdullah Al Noman, Md Fakhruddin, Muttasim Billaha, Moniruzzaman Anik, Tanzil Hasan, Nikhil Tudu, Abdur Rahim, Farzana Mim, Mohammad Jahidur Rahman Khan

**Affiliations:** 1 Department of Microbiology, US-Bangla Medical College and Hospital, Narayanganj, BGD; 2 Department of Gastroenterology and Hepatology, Ehime University Graduate School of Medicine, Toon City, JPN; 3 Molecular Diagnostics Laboratory, Bangabandhu Sheikh Mujib Medical College, Faridpur, BGD; 4 Department of Biochemistry and Molecular Biology, Jahangirnagar University, Dhaka, BGD; 5 Department of Microbiology, Shaheed Suhrawardy Medical College, Dhaka, BGD

**Keywords:** rt-pcr, nasal swab, nasopharyngeal swab, covid-19, sars-cov-2, self-collection

## Abstract

Background: Self-collection of nasal swabs for the detection of SARS-CoV-2 RNA by reverse transcription-polymerase chain reaction (RT-PCR) would considerably increase the testing capability and decrease the risk of transmission among healthcare workers (HCW) and the use of personal protective equipment (PPE).

Objectives: This study aimed to evaluate the performance of self-collected nasal swabs compared with professionally collected nasopharyngeal (NP) swabs for detection of SARS-CoV-2 RNA by RT-PCR.

Materials and methods: We performed a cross-sectional study where the suspected cases of coronavirus disease 2019 (COVID-19) were instructed about the self-collection of nasal swabs from their mid-turbinate. The results were compared to a nasopharyngeal swab collected by a trained healthcare worker in the same patient at the same sitting.

Results: We enrolled 100 participants, of which, 69 (69%) were male and 31 (31%) were female. The median age of the study participant was 36 years. Of the participants, 58 (58%) were symptomatic, and the commonest clinical presentation was cough, which was present in 42 (42%) participants. Out of 100 samples, 31 (31%) professionally collected nasopharyngeal swabs and 28 (28%) self-collected nasal swabs were positive for SARS-CoV-2 by RT-PCR. Out of 31 professionally collected positive samples, three samples were negative in self-collection. Out of 28 self-collected positive samples, no sample was negative in the professional collection. The sensitivity and specificity of self-collected nasal swabs compared to professionally collected nasopharyngeal swabs were 90.32% and 100.00%, respectively. The sensitivity of self-collected nasal was 100% when the cycle threshold (Ct) value of the professionally collected NP swab was less than 30.

Conclusion: Our study showed that self-collected nasal swabs' sensitivities were similar to professionally collected NP swabs with a high viral load (low Ct value). Hence, this method could be used when the patient is symptomatic and come to the health providers in the early stage of COVID-19 illness.

## Introduction

Severe acute respiratory syndrome coronavirus-2 (SARS-CoV-2) that causes coronavirus disease 2019 (COVID-19) has infected over 500 million individuals and caused over six million deaths worldwide. In Bangladesh, the number of SARS-CoV-2 infections is about two million, and the number of death is about 30,000 [1]. Due to mass vaccination, SARS-CoV-2 infections and death are declining worldwide. In Bangladesh, over 11 million people got two doses of the COVID-19 vaccine, about 70% of the total population [2]. The COVID-19 testing rate is decreasing day by day, but the importance of testing remains to conduct epidemiological surveillance to detect possible outbreaks promptly and take necessary actions to prevent disease transmission. Previous experience and several studies suggested that the next pandemic may be caused by a respiratory virus [3]. The sample collection methods influence the test result, whether it is done by reverse transcription-polymerase chain reaction (RT-PCR) or rapid antigen test [4]. Therefore, practicing a feasible sample collection method for respiratory virus infections will play a crucial role in the future pandemic.

Real-time reverse transcription-polymerase chain reaction (qRT-PCR) is the current “gold standard” method to diagnose COVID-19. It requires the collection of a nasopharyngeal (NP) sample from the suspected COVID-19 patient by trained healthcare workers who could engage in other duties [5]. The procedure of NP sampling may be uncomfortable for the patient, discouraging patients from being tested. In addition, the healthcare workers must use personal protective equipment (PPE) during the sample collection as the procedure may produce aerosols containing the SARS-CoV-2 virus from sneezing and coughing resulting from the irritation of the nasopharynx of the patient [6]. However, in many hospitals, such equipment is scarce [7].

Collecting a nasal swab is quicker, more bearable, and creates less chance of coughing, sneezing, and gagging than an NP swab. Earlier studies have suggested that qRT-PCR can detect the SARS-CoV-2 viral RNA efficiently from anterior nasal swab (ANS) and saliva sample (SS) in COVID-19 patients [8,9]. Viral RNA can also be detected from nasal swabs and other upper respiratory infections such as influenza [10-12].

Several studies found a high prevalence of SARS-CoV-2 infection among healthcare workers [13-15]. The prevalence of SARS-CoV-2 infection among the healthcare workers in COVID-19 PCR laboratories is also high. The laboratory workers involved in sample collection possess a greater risk of SARS-CoV-2 infection. Many of them become the source of laboratory cross-contamination [16]. If the self-collected nasal swab can be established as a dependable alternative to professionally collected swabs, it would decrease the infection rate among healthcare workers and biosafety risk. It will also increase the testing capacity and help preserve PPE. In this study, we evaluate the performance of self-collected nasal swabs compared with professionally collected nasopharyngeal swabs to detect SARS-CoV-2 RNA by the RT-PCR method.

## Materials and methods

Study population

The Ethical Review Board of US-Bangla Medical College and Hospital, Narayanganj, Bangladesh, approved this study (serial number-USBCH/Ethical/2020/01). This cross-sectional study was conducted at Gazi COVID-19 PCR Laboratory, Narayanganj, Bangladesh, from August 2020 to February 2021. This study included the patients who came to this laboratory with the clinical symptom of COVID-19 or with a history of contact with COVID-19 patients.

Specimen collection

The participants were supplied with a swab kit that included a flocked swab with nylon microfibers (Shenzen, China: J. Able Technology Co.) and a 1 ml tube with sample storage reagent (Hunan, China: Sansure Biotech Co., Ltd.). A printed set of instructions, including images, explained how to collect nasal swabs. Participants were instructed to insert the absorbent tip of the swab within the nostril of approximately 1.5 cm for 10 seconds, turn and swirl the swab twice at least while removing the swab slowly, and then place the cotton tips inside the tube filled with viral transport medium (VTM) and break the stick from the breaking point before securely closing the tube cap. A trained health care worker was present with the participant during the procedure. Immediately after the self-collection, the health care worker took a nasopharyngeal (NP) sample from the other nostril of the participant. Both samples were labeled and preserved at 2-8°C. We took informed written consent from the participant and filled out a data collection sheet before collecting samples.

Real‐time RT‐PCR assays for the detection of SARS‐CoV‐2 RNA

We used Sansure Novel Coronavirus (2019-nCoV) Nucleic Acid Diagnostic Kit (PCR-Fluorescence Probing; Hunan, China: Sansure Biotech Co., Ltd.) for detecting SARS-CoV-2, which targeted two SARS‐CoV‐2 genes, namely, the ORF1ab and N genes. Quant Studio 5 (Applied Biosystems; Waltham, MA: Thermo Fisher Scientific) was used for genome amplification. There was a valid internal control (IC) which is <40 cycle threshold (Ct) value, and a sample was reported to test positive for SARS-CoV-2 if any of the two genes, ORF 1ab (Fam) and N gene (ROX) or both genes showed Ct value less than 40.

Data analysis

The performance of self-collection of the sample was assessed, calculating its sensitivity and specificity. Sample collection by trained health care workers was considered the "gold standard" for calculating the positivity or negativity of the samples. SPSS version 26.0. (Chicago, IL: SPSS Inc.) was used to analyze all data.

## Results

A total of 100 participants were included in this study, of which, 69 were male and 31 were female, with a median age of 36 years. In addition, 58 (58%) participants were symptomatic, and the commonest clinical presentation was cough which was present in 42 (42%) participants. The demographic and clinical profiles of the patients have been shown in Table 1.

**Table 1:** Demographic and clinical data of all participants.

Characteristics	Total	Professionally collected		Self-collected	
		Positive	Negative	Positive	Negative
Sample number (%)	100 (100)	31 (31)	69 (69)	28 (28)	72 (72)
Age (years), median	36	36	36	36	36
<18 (%)	1 (1)	0 (0)	1 (1.45)	0 (0)	1 (1)
18-64 (%)	91 (91)	28 (90)	63 (91.30)	25 (89)	66 (92)
>64 (%)	8 (8)	3 (10)	5 (7.25)	3 (11)	5 (7)
Sex					
Male (%)	69 (69)	24 (86)	45 (65)	21 (75)	48 (67)
Female (%)	31 (31)	7 (23)	24 (35)	7 (25)	24 (33)
Clinical features					
Symptomatic patients (%)	58 (58)	24 (86)	34 (49)	22 (79)	36 (50)
Asymptomatic patients (%)	42 (42)	7 (23)	35 (51)	6 (21)	36 (50)
Fever	34 (34)	13 (38)	21 (62)	11 (23)	23 (68)
Cough	42 (42)	17 (40)	25 (60)	15 (36)	27 (64)
Sore throat	17 (17)	9 (53)	8 (47)	9 (53)	8 (47)
Difficulty in breathing	7 (7)	6 (86)	1 (14)	6 (86)	1 (14)
Loose motion	1 (1)	1 (100)	0 (0)	1 (100)	0 (0)

Out of 100 samples, 31 professionally collected nasopharyngeal (NP) swabs and 28 self-collected nasal swabs were reported positive for SARS-CoV-2 by RT-PCR. Out of 31 professionally collected positive samples, three samples were negative in self-collection. Out of 28 self-collected positive samples, no sample was negative in collection with professionals. The sensitivity and specificity of self-collected nasal swabs compared to professionally collected NP swabs were 90.32% and 100.00%, respectively. The positive predictive value and negative predictive value of self-collected nasal swabs compared to professionally collected NP swabs were 100.00% and 95.83%, respectively (Table 2).

**Table 2:** The sensitivity and specificity of self-collected nasal swabs compared with professionally collected NP swabs among the participants. NP: nasopharyngeal

Characteristics	Professionally collected positive (%)	Professionally collected negative (%)	Total (%)
Self-collected positive (%)	28 (28%)	0 (0%)	28 (28%)
Self-collected negative (%)	3 (3%)	69 (69%)	72 (72%)
Total (%)	31 (31%)	69 (69%)	100 (100%)
Sensitivity (%)	90.32% (74.25-97.96%)		
Specificity (%)	100.00% (94.79-100.00%)		
Positive predictive value (%)	100.00%		
Negative predictive value (%)	95.83% (88.70-98.54%)		

**Figure 1:**
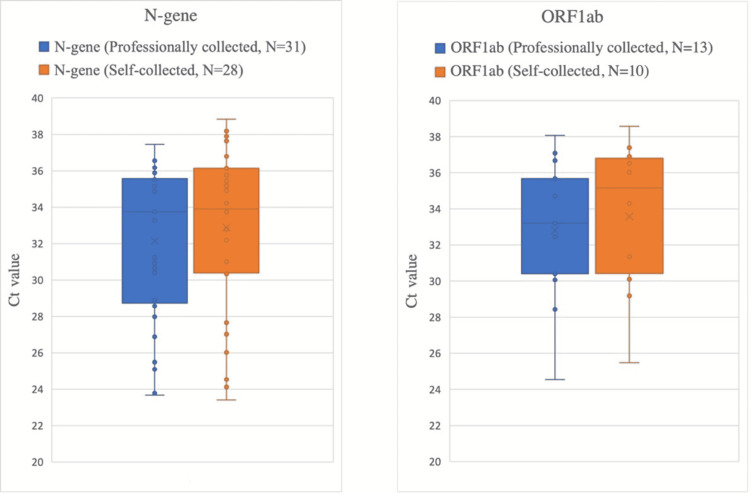
Comparison of Ct values of all samples tested positive for N-gene and ORF1ab. Ct values: cycle threshold values

Ct-values of all samples that tested positive for N-gene and for ORF1ab are presented in Figure 1. In professionally collected samples, median Ct-values were 32.14 for the N-gene and 32.80 for ORF1ab. Median Ct-values were 32.88 for N-gene and 33.57 for ORF1ab in self-collected samples.

**Figure 2:**
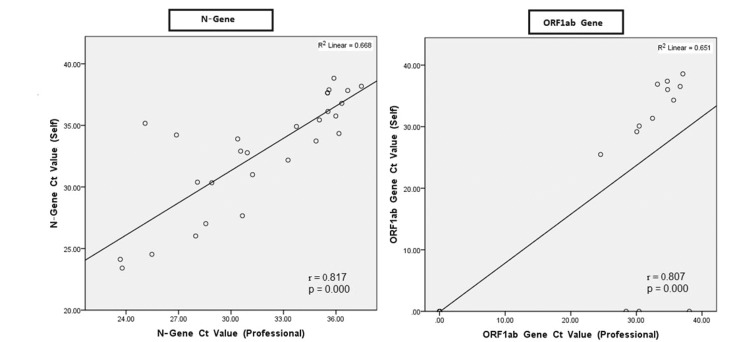
Ct-values of every self-collected nasal swab compared with the Ct-value of the professionally collected NP sample, expressed for the two target genes. Ct values: cycle threshold values; NP: nasopharyngeal

Figure 2 presents N-gene and ORF1ab gene, Ct-values of both sampling methods were markedly correlated with their respective Ct-values for the NP (p<0.001 for all comparisons), with the highest correlation for the N-gene (r = 0.817). The sensitivity of self-collected nasal swab, stratified by Ct values of professionally collected NP swab is presented in Table 3.

**Table 3:** Sensitivity of self-collected nasal swab, stratified by Ct values of professionally collected NP swab. Ct values: cycle threshold values; NP: nasopharyngeal

Ct values of professionally collected NP swab	Number of participants	Sensitivity
<25	2	100% (15.8-100)
25-30	22	100% (84.6-100)
>30	7	70% (34.8-93.3)

## Discussion

Sensitive diagnosis of COVID-19 is vital for patient management, prevention of hospital-acquired infection, and control of any public health emergency. The collection of clinical samples in the correct technique is crucial in performing diagnostic tests, including nucleic acid amplification (NAAT) methods. Self-collection of respiratory samples for viral infections is not new [17-19]. Centers for Disease Control and Prevention (CDC) and the Infectious Diseases Society of America (IDSA), both recommend the use of NP swab or nasal swab (either professionally or self-collected) for the diagnosis of SARS-CoV-2 infection [20,21]. In another study, self-collected nasal swabs were found to be a dependable alternative to professionally collected NP swab for detecting influenza A and B viruses by RT-PCR [22]. Another study presented similar findings with several respiratory viruses [23]. Despite this, data from the performance of distinct types of samples from the same person simultaneously by NAAT methods are still not adequate.

Our study shows that the sensitivity of a self-collected nasal swab is less than the professionally collected NP swab. Another notable finding is that the self-collected nasal swabs provide higher sensitivity when the viral load is higher, commonly found in the early stages of SARS-CoV-2 infection. The sensitivity of self-collected nasal swabs remarkably decreased when the Ct values of the professionally collected swab exceeded 30, which is also similar to findings of previous studies (Table 3) [24-26]. Another study revealed that the viral cultures became negative when the Ct value of a sample was over 30 (when viral load is low) [27]. According to the finding of another study, the SARS-CoV-2 virus frequently cannot be cultured or isolated after 11 days of symptom onset [28]. Hence, the findings of this study encourage the self-collection of nasal swabs instead of professionally collected NP swabs during the early days of SARS-CoV-2 infection when viral loads are high, and the sensitivity of self-collection of nasal swabs are similar to professionally collected NP swab.

We prefer supervised self-collection in healthcare center over unsupervised self-collection at patient’s home. Extensive and unsupervised application of self-collection methods can reduce the quality of testing. The consistency and efficiency of self-collection methods may depend on social and economic conditions, thus influencing the test performance. A traveler who needs a COVID-19 negative test result for immigration clearance or a person who faces unemployment or economic loss if tested positive may intentionally perform a faulty self-test to affect the test result [29]. In our study, a well-trained medical technologist was always present when the participant performed self-collection. Despite clear instructions, many participants still required help during self-collection. The most frequent problem was that they needed direction in breaking the swab stick. A prior study mentioned the problem of difficulty in breaking the swab stick as we faced in our case [30]. So, a trained healthcare worker should be present (beyond three feet) at self-collection to troubleshoot and confirm that all the steps are carried out properly.

Self-collection provides several advantages to both patients and healthcare workers. Patients usually prefer nasal swab over NP swab. Collection of NP swabs can trigger sneezing and coughing which may be awkward for the patient and raise the risk of SARS-CoV2 transmission by aerosol to healthcare workers. In addition, self-collected sampling decreases personal protective equipment (PPE) use, which is short in supply during the pandemic. It also increases the quality of the health care system by optimizing staff utilization [30].

This study has some limitations. The sample size was comparatively small, and only self-collected nasal swabs and professionally collected NP swabs were compared due to the work overload, shortage of manpower, and limited testing kits. The study was conducted in a single institution where patients attended from a particular area.

## Conclusions

Supervised self-collected nasal swabs delivered a similar performance to professionally collected NP swabs when the viral load was high. This method was feasible, especially for symptomatic patients, and can expand the testing capacity by removing the requirement for a healthcare worker to collect each sample, decreasing the risk of transmission, and optimizing PPE use for testing.
